# Coverage and determinants of second-dose measles vaccination among under-five children in East Africa countries: a systematic review and meta-analysis

**DOI:** 10.3389/fpubh.2024.1359572

**Published:** 2024-05-01

**Authors:** Tewodros Getaneh Alemu, Tadesse Tarik Tamir, Belayneh Shetie Workneh, Enyew Getaneh Mekonen, Mohammed Seid Ali, Alebachew Ferede Zegeye, Mulugeta Wassie, Alemneh Tadesse Kassie, Berhan Tekeba, Almaz Tefera Gonete

**Affiliations:** ^1^Department of Pediatrics and Child Health Nursing, School of Nursing, College of Medicine and Health Sciences, University of Gondar, Gondar, Ethiopia; ^2^Department of Emergency and Critical Care Nursing, School of Nursing, College of Medicine and Health Sciences, University of Gondar, Gondar, Ethiopia; ^3^Department of Surgical Nursing, School of Nursing, College of Medicine and Health Sciences, University of Gondar, Gondar, Ethiopia; ^4^Department of Medical Nursing, School of Nursing, College of Medicine and Health Sciences, University of Gondar, Gondar, Ethiopia; ^5^School of Nursing, College of Medicine and Health Sciences, University of Gondar, Gondar, Ethiopia; ^6^Department of Clinical Midwifery, School of Midwifery, College of Medicine and Health Sciences, University of Gondar, Gondar, Ethiopia

**Keywords:** children, coverage, East Africa, immunization measles, second dose, vaccination

## Abstract

**Background:**

One of the biggest breakthroughs of contemporary medicine is measles vaccination. It is essential for the total elimination of measles. Understanding the magnitude and determinants of effective second-dose measles vaccination coverage is a critical task. Accordingly, we set out to check the best available evidence of the pooled second-dose measles vaccination coverage among under-five children in East Africa.

**Method:**

We searched electronic databases such as PubMed, Google Scholar, Cochrane, and others. Two reviewers separately carried out the search of the Joanna Briggs Institute, selection of studies, critical appraisal, and data extraction. A third party was involved in resolving the disagreement among the reviewers. Seven studies included in this study, four from Ethiopia, two from Kenya, and one from Tanzania were cross-sectional and published in English language, with publication dates before 29 November 2023. Articles lacking full-text, the intended outcome, and that are not qualitative studies were excluded from the analysis. The Microsoft Excel checklist was used to extract the data and then exported to STATA 11. In addition, *I*^2^, Funnel plots, and Egger's test were employed to measure heterogeneity and detect publication bias, respectively. A random effect model was used.

**Result:**

The meta-analysis includes a total sample size of 4,962 children from seven articles. The pooled prevalence of second-dose measles vaccination among under-five children in East Africa was found to be 32.22% [95% CI; (18.82, 45.63)], and the significant factors were as follows: birth order (1.72; OR = 95% CI: 1.32, 2.23), information about measles-containing second-dose vaccine (MCV 2) (7.39; OR = 95% CI: 5.21, 10.50), mother's marital status (1.47; OR = 95% CI: 1.05, 2.07), complete immunization for other vaccines (2.17; OR = 95% CI: 1.49, 3.17), and distance of vaccination site (3.31; OR = 95% CI: 2.42, 4.53).

**Conclusion:**

The current study found that pooled prevalence of second-dose measles vaccination coverage among under-five children was still very low. It was also observed that birth order, distance of the vaccination site, complete immunization for other vaccines, mother's marital status, and information about MCV were factors associated with second-dose measles vaccination. These factors imply that there is a need for countries and their partners to act urgently to secure political commitment, expand primary health service and health education, and increase vaccination coverage.

## Introduction

Measles is a highly contagious virus that can result in serious illness, lifelong problems, and fatalities (1). The first dose of the measles-containing vaccine should be given to infants as early as 9 months of age in nations where the disease is still spreading, and the second dose should be given as late as 15–18 months (2). The World Health Organization (WHO) recommends that two doses of the measles-containing vaccine (MCV) be included in all national immunization regimens. An estimated 169 million children worldwide are believed to have missed out on receiving the first dose of the measles vaccine between 2010 and 2017 and an additional 19.2 million in 2018 (3, 4). Furthermore, measles led to a loss of 140,000 lives worldwide in 2018, according to estimates from the United States Centers for Disease Control and Prevention and WHO (4). Countries in all the six WHO regions have adopted measles elimination goals (5). The elimination of measles is confirmed by the absence of endemic measles transmission in a region or other defined geographical area for a minimum of 1 year within the framework of an efficient surveillance system. Between 2000 and 2015, there was a 70% decline in the global number of recorded cases of measles, from 853,479 to 254,928, and a 75% fall in the incidence of measles cases per million people, from 146 to 36. These patterns show progress toward both regional and global measles elimination targets as well as milestones for measles control (3, 6). Moreover, WHO, UNICEF, and other partners created the Global Measles and Rubella Strategic Plan 2012–2020 (7). This strategy plan's primary goal was to provide the measles-containing second-dose vaccine (MCV2) to every child (8). However, none of the 2020 milestones or elimination goals (less than one case per 100,000 population per year) were met (9). Some nations still experience repeated outbreaks of measles despite the UNICEF and WHO's comprehensive measles reduction strategy, as well as the cooperation of international organizations for reducing mortality due to measles (3). The vaccination of at least 95% of the population with two doses of the measles vaccine effectively prevents the incidence and transmission of the disease within that community, ensuring herd immunity and the protection of all individuals, including those who are not vaccinated (10). MCV2 coverage in the WHO European Region was just 90% (11). Although MCV2 has recently been introduced in Africa, most nations still have minimal coverage. Of the 26 nations that implemented MCV2, only eight achieved a coverage rate of above 80% in 2015 (5). In seven nations, the coverage ranged from 60 to 80%, while in eight countries, it was < 60% (5). Nonetheless, a great number of people die due to the highly contagious measles every year (12). An estimated 207,500 measles deaths were reported worldwide in 2019, with 147,900 (more than 70%) of those deaths occurring in African nations (12). Over the past 10 years, there has been a decrease in the death rate due to measles in Africa (13); however, the disease remains an issue in the region (14, 15). Although some studies have reported the determinants of second-dose measles vaccination coverage in East Africa, none of them have systematically reviewed the second-dose measles vaccination coverage, which varies and is not uniform throughout the nation. Public health stakeholders must choose the optimal vaccination schedules based on their nation's epidemiology, the features of its health system, and the best available data regarding the second-dose measles vaccination coverage at measles elimination in order to control the disease. The reported determinants include antenatal care (ANC), mother's education, place of delivery, birth order, receiving pentavalent 3, age of the child, information about MCV2, distance of the vaccination site, knowledge about immunization, attitude, maternal age, complete immunization, postnatal check, waiting time, residence near the health facilities, family size, household wealth status, maternal occupation, and mother's marital status (16–18). Thus, the current study aims at identifying relevant studies and summarizing major determinants of second-dose measles vaccination coverage in East Africa. The results of this review will add to existing knowledge about the problem and guide policymakers to improve second-dose measles vaccination coverage in East Africa.

## Method and materials

### Searching strategy and data source

All published studies conducted in East Africa reporting the second-dose measles vaccination coverage from September 2016 to 2022 were included. Only cross-sectional, human, and English language research were included in the search parameters. The Preferred Reporting Items for Systematic Review and Meta-Analysis statement (PRISMA) guidelines were followed in reporting the review's findings (19). To get the relevant articles, PubMed, Cochrane, Google Scholar, and other electronic databases were accessed. Furthermore, articles were searched by looking through the reference lists of previously recognized articles as well as the gray literature that was available in the repository of the local university. The article search was conducted independently and systematically by the authors. Furthermore, a manual cross-referencing search of the gray literature was conducted to locate additional noteworthy articles. The core search terms and phrases were “Child,” “Children,” “Coverage,” “Second Dose Measles,” “Vaccination,” “magnitude of Second Dose Measles coverage,” “associated factors,” “Immunization Coverage,” and “East Africa.” We used various Boolean operators to construct search algorithms for the Medical Subject Headings (MeSH terms) below. Particularly, to fit advanced PubMed database, the following search strategy was applied: (((((((((Epidemiologic) OR (Child)) OR (Children)) AND (Coverage, Second Dose Measles)) OR (Second Dose Measles coverage)) OR (Coverage, Vaccination)) OR (Vaccination coverage)) OR (Immunization Coverage)) OR (Coverage, Immunization)) AND (East Africa).

### Inclusion and exclusion criteria

Those studies included in this systematic review and meta-analysis were the studies with the prevalence and/or at least one associated factor of second-dose measles vaccination coverage, studies conducted in East Africa, studies published in English language, and studies published before 29 November 2023. Unpublished studies, book reviews, and case reports, publications with only an abstract, studies that did not identify the intended outcome, qualitative studies, and studies conducted outside East Africa were excluded.

### Types of exposure

To evaluate the effects on second-dose measles vaccine coverage, factors influencing such coverage were taken into account as exposure variables in this systematic review and meta-analysis.

### Outcome of interest

The second-dose measles vaccination coverage was calculated by dividing the number of children who received a second dose of the measles vaccination by the total number of children involved in the research and then multiplying the result by 100. Mothers' verbal reports and/or immunization cards were used in studies that were included in this systematic review and meta-analysis to ascertain whether or not a child received the vaccine. The identified predictors were antenatal care (ANC) (< 4, and ≥4), mother's education (formal education and non-formal education), place of delivery (health facility vs. home), birth order (first vs. two and above), received pentavalent 3 (yes vs. no), information about MCV2 (yes vs. no), distance to the vaccination site ( ≤ min and >30 min), knowledge about immunization (yes vs. no), attitude (good vs. poor), complete immunization (yes vs. no), postnatal check (yes vs. no), waiting time (< 1 and ≥1 h), residence (urban vs. rural), family size ( ≤ 5 and >5), household wealth status (rich vs. poor), maternal occupation (employed vs. unemployed), and marital status (married vs. unmarried).

### Study selection

The authors TGA and ATG conducted an initial search across several databases in order to eliminate duplicate studies. The retrieved studies were exported to the reference manager program, Endnote version 9. The titles and abstracts of the research were checked and evaluated by the same two authors (TGA and ATG), who then independently evaluated the full texts. Disagreements were resolved by consensus.

### Methods of data extraction and quality assessment

All studies that were accepted based on the full-text screening were retained for data extraction.

A data extraction form was developed, which the authors TGA and BT then used for extracting data from each of the included studies. To retrieve the data, a standardized data extraction form for Microsoft Excel was used. Significant information was acquired from the included studies, including the first author's name, the year of publication, the study location, the nations under investigation, the study design, associated variables, sample size, the number of outcomes, the prevalence (magnitude), the risk estimate (odds ratio), and 95% confidence interval (CI). A quality appraisal checklist from the Joanna Briggs Institute (JBI) was used to assess the quality of the included studies. Cross-sectional studies were evaluated using the following eight criteria: inclusion criteria, study subject and setting description, valid and reliable exposure measurement, objective and standard criteria applied, confounder identification, confounder handling strategies, outcome measurement, and appropriate statistical analysis. When a study achieved a quality assessment indicator score of 75–100%, it was considered high quality, a score of 50–74% indicated moderate quality, and a score of 0–49% represented low quality. These indicators resulted in six studies rated as high quality and one as moderate quality (Table 1).

**Table 1:** Characteristics and quality status of the studies included to assess the pooled magnitude of second-dose measles vaccination coverage in East Africa.

**ID**	**First author**	**Year of publication**	**Country**	**Study design**	**Study population**	**Sample size**	**Number of outcome**	**Prevalence**	**Quality status**
1	Joseph Obiero Ogutu, et al.	2020	Kenya	Cross-sectional	Children aged 19–59 months	417	213	51.08	Low risk
2	Atalay Goshu Muluneh, et al.	2019	Ethiopia	Cross-sectional	Children aged < 36 months	965	120	12.44	Low risk
3	Aynalem Demewoz, et al.	2020	Ethiopia	Cross-sectional	Children aged 24–35 months	837	403	48.15	Low risk
4	Fredrick Mike Makokha, et al.	2016	Kenya	Cross-sectional	Children aged 24–35 months	571	102	17.86	Low risk
5	Richard Magodi	2017	Tanzania	Cross-sectional	Children aged < 5 years	1,000	442	44.20	Low risk
6	Addisu Waleligne Tadesse, et al.	2022	Ethiopia	Cross-sectional	Under-five children	372	158	42.47	Low risk
7	Achamyeleh Birhanu Teshale, et al.	2019	Ethiopia	Cross-sectional	Children aged 24–35 months	800	79	9.88	Low risk

### Data processing and analysis

Pooled analysis was conducted using weighted inverse variance random-effects model (20). For the meta-analysis, STATA version 11 statistical software was employed. The funnel plot and Egger's regression test were used to more objectively assess publication bias (21). The studies' heterogeneity was measured using the I-squared statistic; An *I*-squared statistic of 25, 50, and 75%, respectively, indicated low, moderate, and high heterogeneity (22, 23). Sensitivity analysis was used to see how one study affected the estimate as a whole. To determine the relationship between determinant factors and outcome variables in the included articles, the odds ratio was employed.

## Results

### Searching results

**Figure 1:**
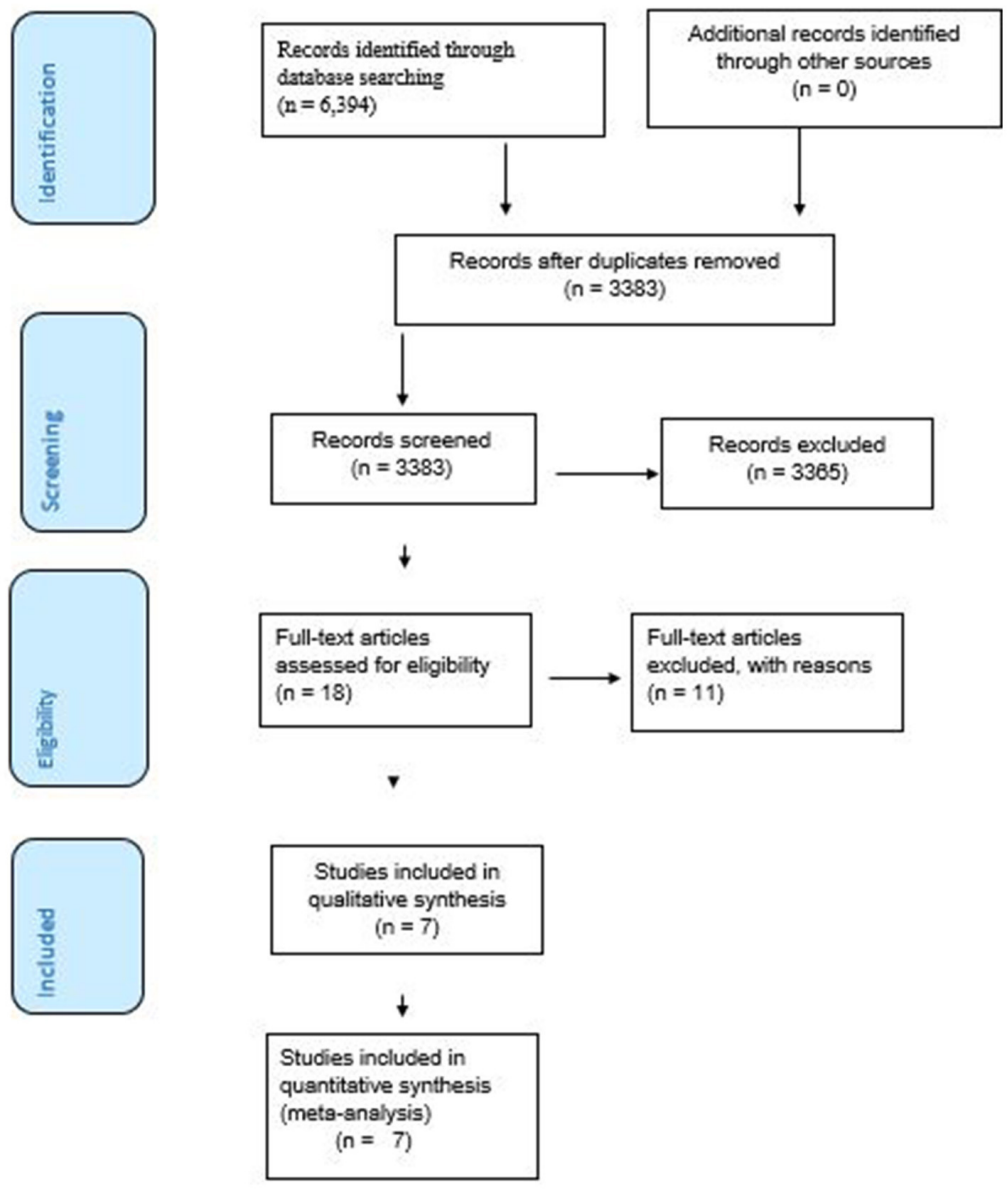
A PRISMA flow diagram of articles screening and process of selection.

The search strategy retrieved 15 articles from Cochrane library, 19 from Pub Med, and 6,360 from Google Scholar. After retrieval, 3,011 articles were removed as they were duplicates, 3,239 due to outcomes mixed with other non-relevant indicators, and 126 due to study area. A total of 18 articles were selected for full-text review. Out of them, 11 articles that failed to provide the outcome of interest were removed from the analysis following full-text reviews. Finally, this systematic review and meta-analysis comprised seven articles to determine the coverage of second-dose measles vaccination and associated factors in East Africa (Figure 1).

### Characteristics of the included studies

Four studies were found in Ethiopia (17, 18, 24, 25), two in Kenya (16, 26), and one in Tanzania (27). All the seven studies employed a cross-sectional study design. Regarding the year of publication, four studies were published before 2020 and three studies were published between 2020 and 2022 (Table 1).

### Magnitude of second-dose measles vaccination coverage in East Africa

**Figure 2:**
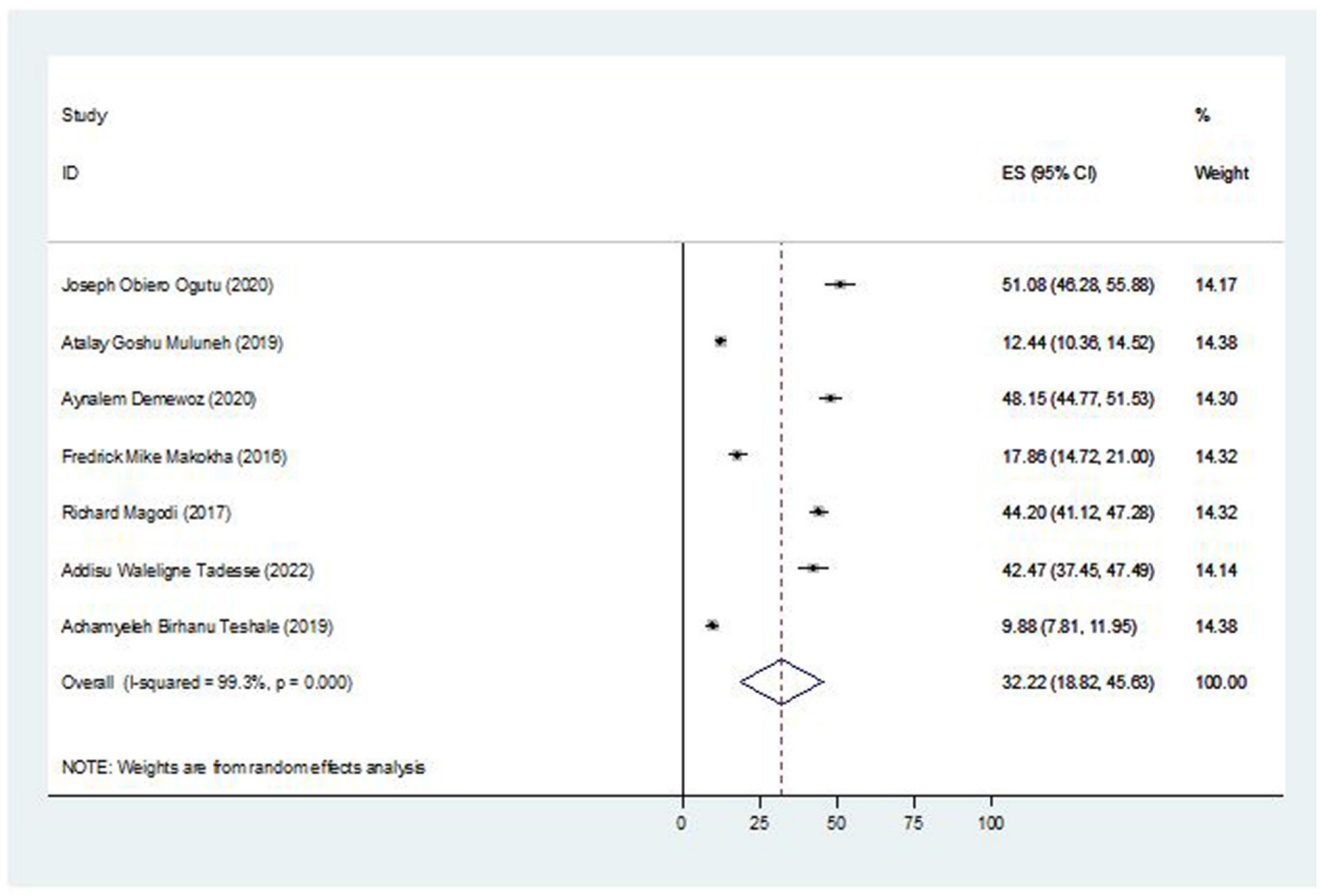
Forest plot of the pooled prevalence of second-dose measles vaccination coverage in East Africa, 2023.

The pooled prevalence of second-dose measles vaccine coverage in East Africa was estimated by a meta-analysis encompassing seven studies with a total of 4,962 participants. Consequently, the overall pooled prevalence of second-dose measles vaccination coverage in East Africa was 32.22% [95% CI; (18.82, 45.63); *I*^2^ = 99.3% (Figure 2)].

### Subgroup analysis

**Figure 3:**
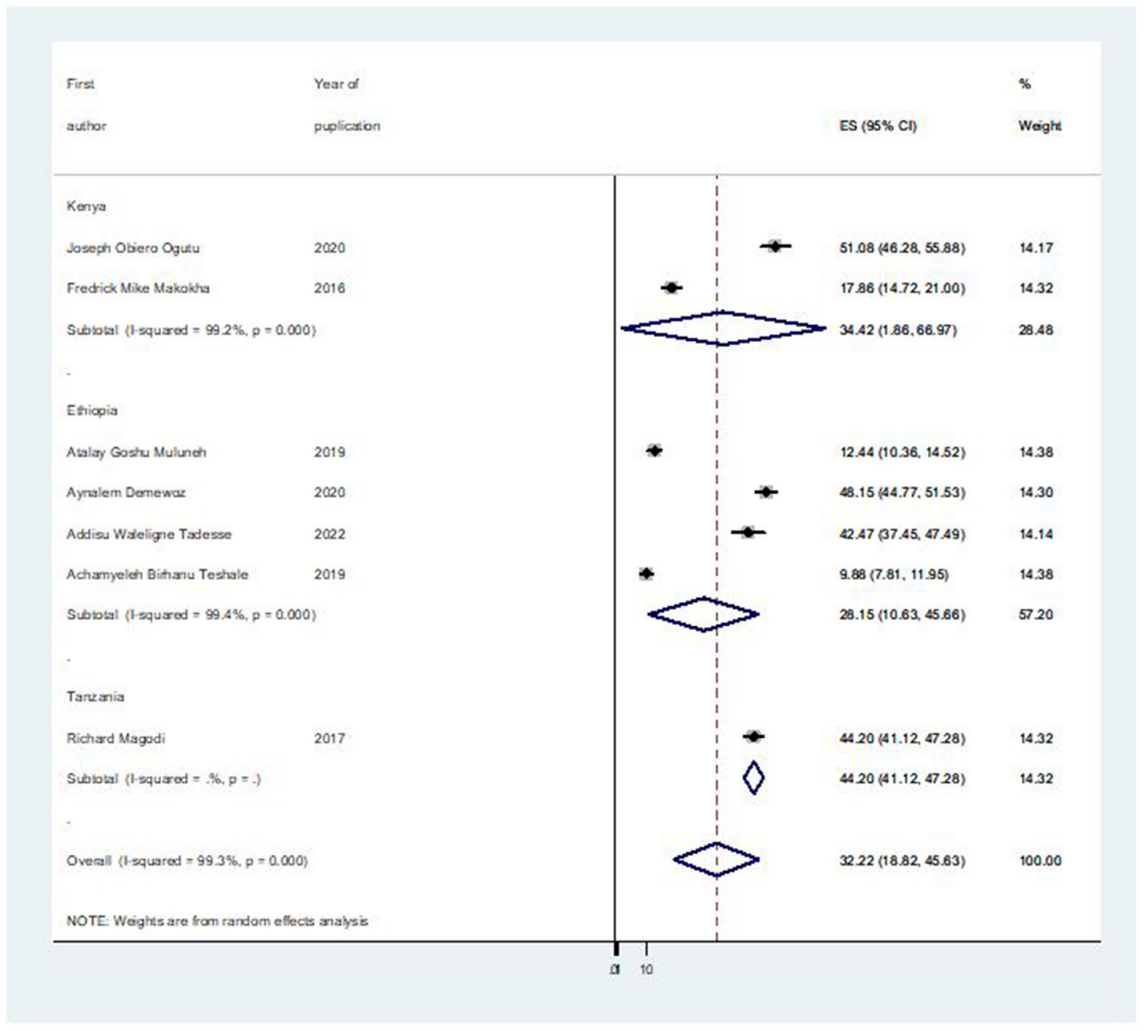
Forest plot of the subgroup prevalence of second-dose measles vaccination coverage in East Africa, 2023.

Based a country-based subgroup analysis, Tanzania had the highest prevalence of second-dose measles vaccination coverage of 44.20% (95% CI: 41.12, 47.28), followed by Kenya at 34.42% (95% CI: 1.86, 66.97) (Figure 3).

### Publication bias

**Figure 4:**
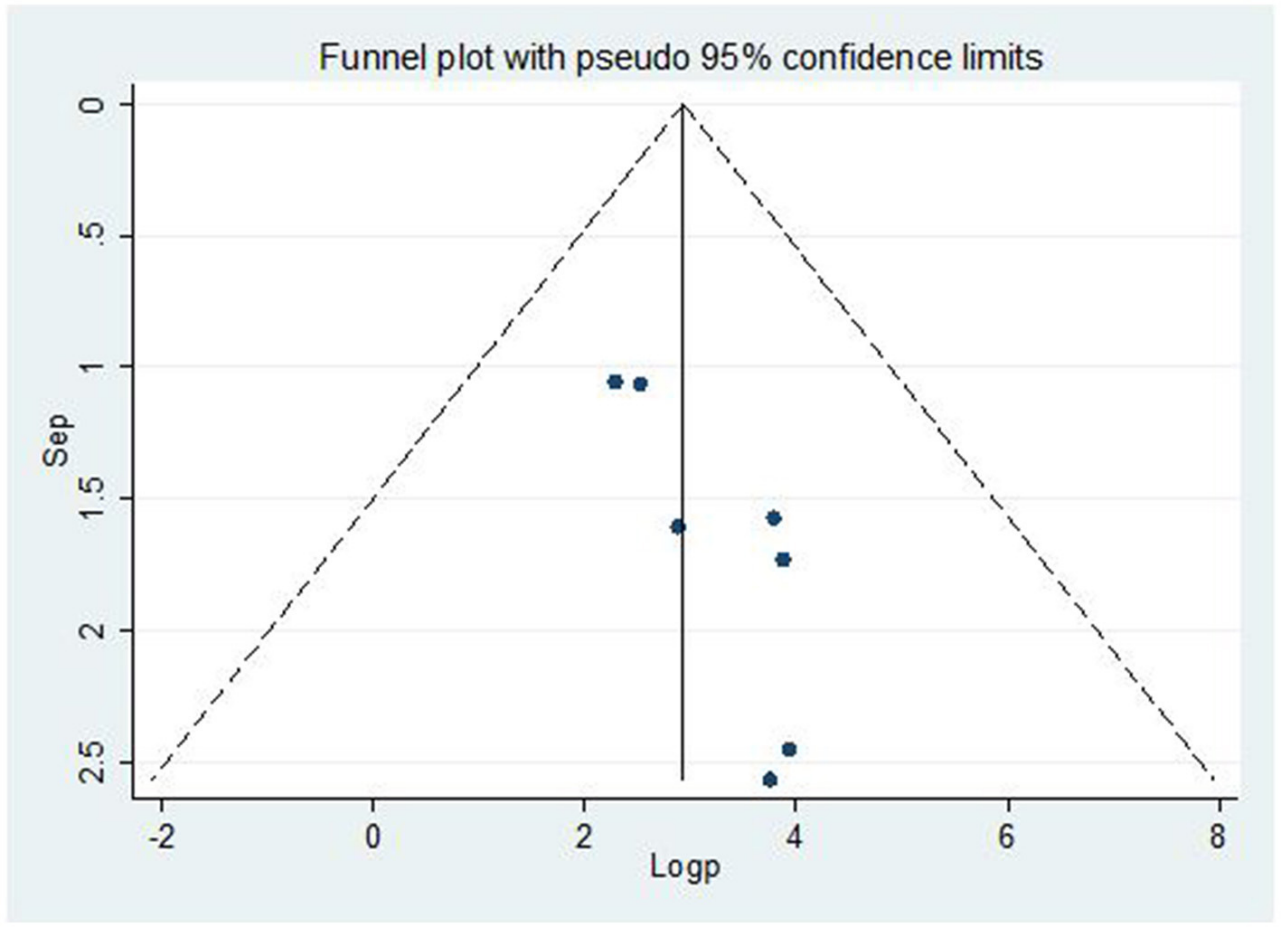
Funnel plot for publication bias, Log prop, or log of proportion (LNP) represented in the *x*-axis and standard error of log proportion in the *y*-axis.

The Egger's regression test and a funnel plot were used to assess publication bias. Subjectively, a funnel plot with an uneven distribution (Figure 4) suggests the existence of publishing bias. In addition, the objective *p*-value of 0.019 from the Egger's regression test indicated the existence of publication bias.

### Sensitivity analysis

**Figure 5:**
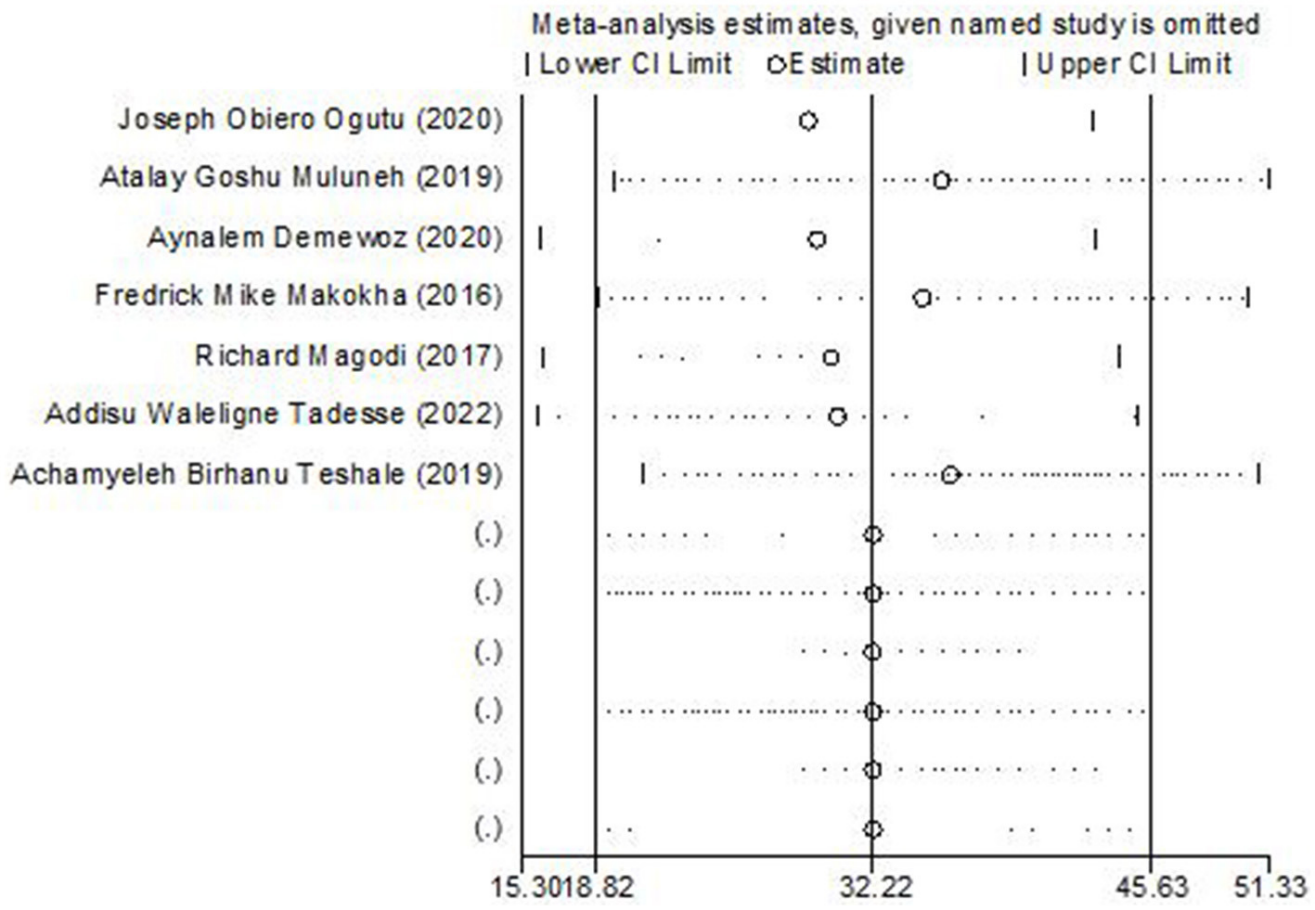
Sensitivity analysis of the included studies.

To determine the weight of each study on the aggregated effect size of magnitude of second-dose measles vaccine coverage, we performed a sensitivity analysis. The Der Simonian-Laird random-effects model sensitivity analysis revealed that no single study had an impact on the overall magnitude of second-dose measles vaccination coverage in East Africa (Figure 5).

### The association between birth order and second-dose measles vaccination coverage

**Figure 6:**
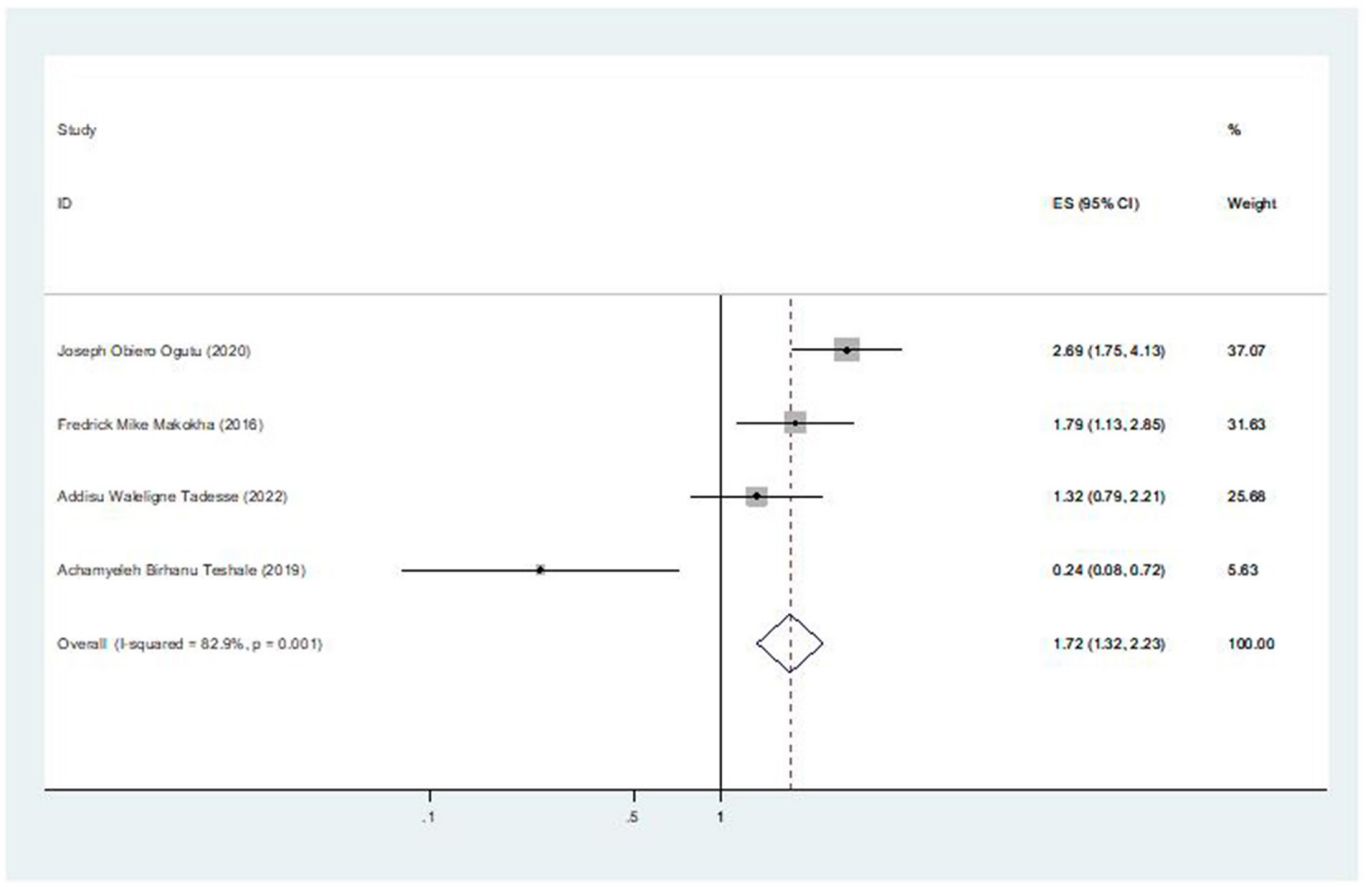
The pooled effect of birth order on second-dose measles vaccination coverage in East Africa.

Among the included seven studies, four studies reported the association between birth order and second-dose measles vaccination coverage. The pooled odds ratio from these studies was 1.72 (95% CI: 1.32, 2.23), which revealed that under-five children with birth orders larger than one were 1.72 times more likely than their counterparts to receive the second dose of the measles vaccination (Figure 6).

### The association between information about MCV2 and second-dose measles vaccination coverage

**Figure 7:**
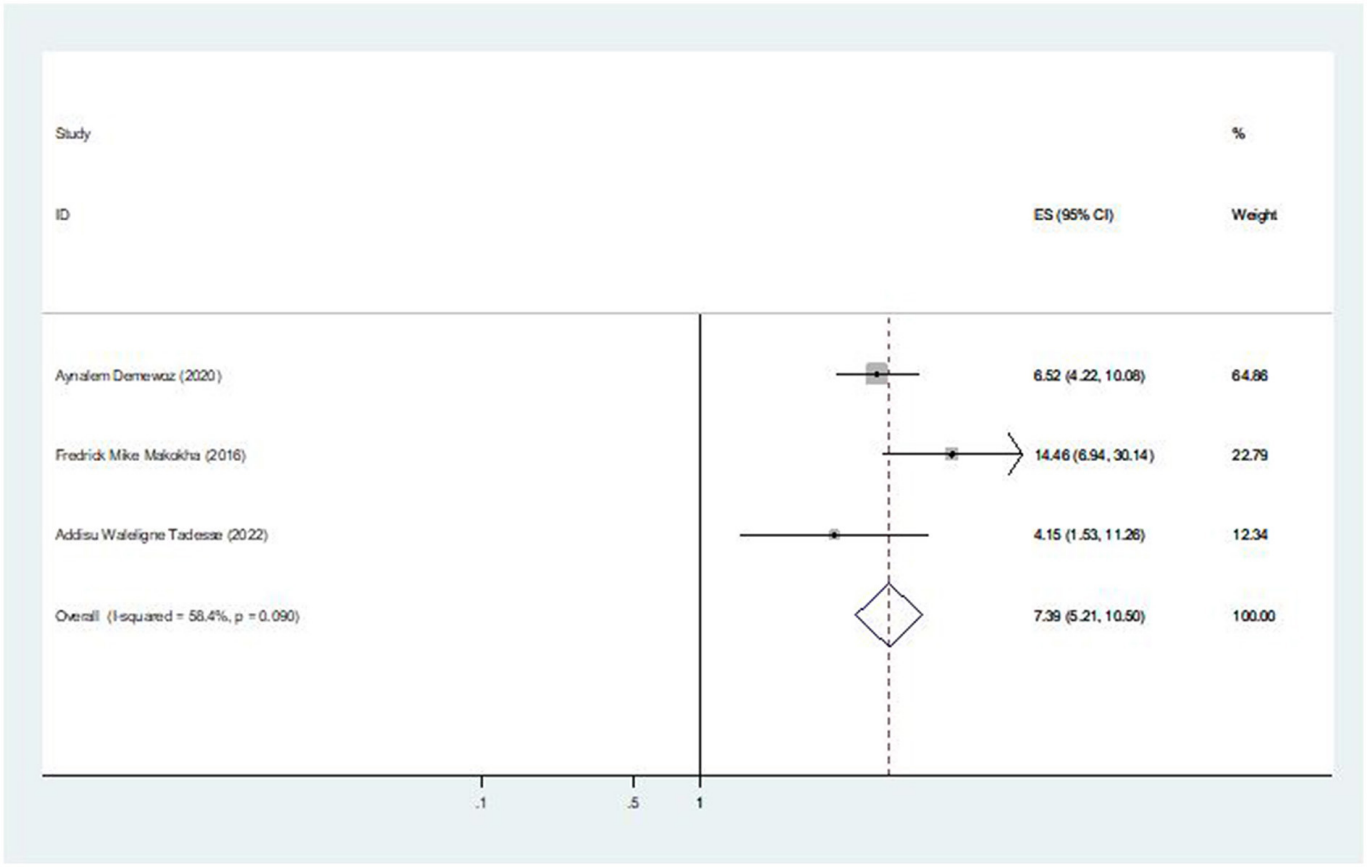
The pooled effect of information about MCV2 on second-dose measles vaccination coverage in East Africa.

Three of the seven included studies revealed an association between coverage of the second dose of the measles vaccination and information of MCV2. The pooled odds ratio was 7.39 (95% CI: 5.21, 10.50), indicating that mothers who were aware of the second dose of the measles vaccine were 7.39 times more likely to vaccinate their children than those who were unaware of the second-dose measles vaccination (Figure 7).

### The association between marital status and second-dose measles vaccination coverage

**Figure 8:**
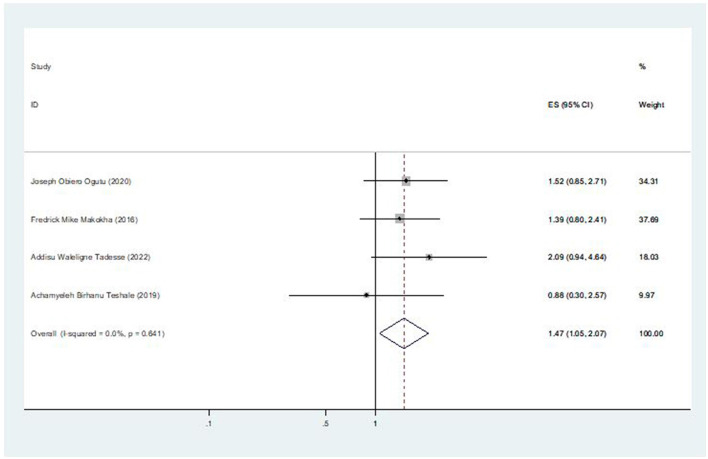
The pooled effect of marital status on second-dose measles vaccination coverage in East Africa.

Four of the seven included studies revealed an association between the coverage of second-dose measles vaccination and mother's marital status. The pooled odds ratio was 1.47 (95% CI: 1.05, 2.07), indicating that children from married women are 1.47 times more likely to receive the second dose of the measles vaccination than children from unmarried women (Figure 8).

### The association between complete immunization for other vaccines and second-dose measles vaccination coverage

**Figure 9:**
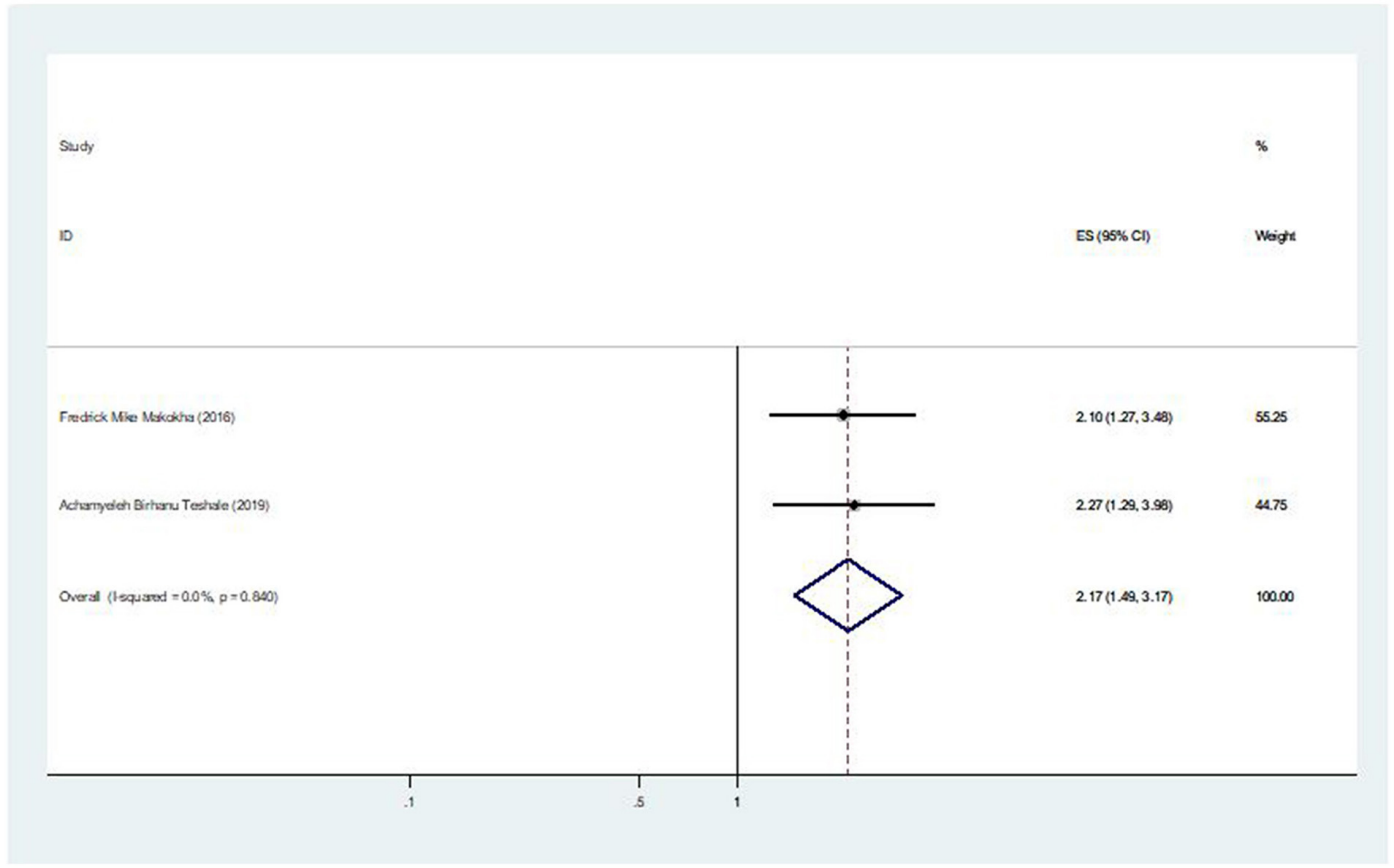
The pooled effect of complete immunization for other vaccines on second-dose measles vaccination coverage in East Africa.

Two of the seven included studies revealed an association between the coverage of second-dose measles vaccination and complete immunization for other vaccines. The pooled odds ratio was 2.17 (95% CI: 1.49, 3.17), indicating that children who had received all other recommended vaccinations were 2.17 times more likely to receive the second dose of the measles vaccine than children who had not received all other recommended vaccinations (Figure 9).

### The association between distance of vaccination site and second-dose measles vaccination coverage

**Figure 10:**
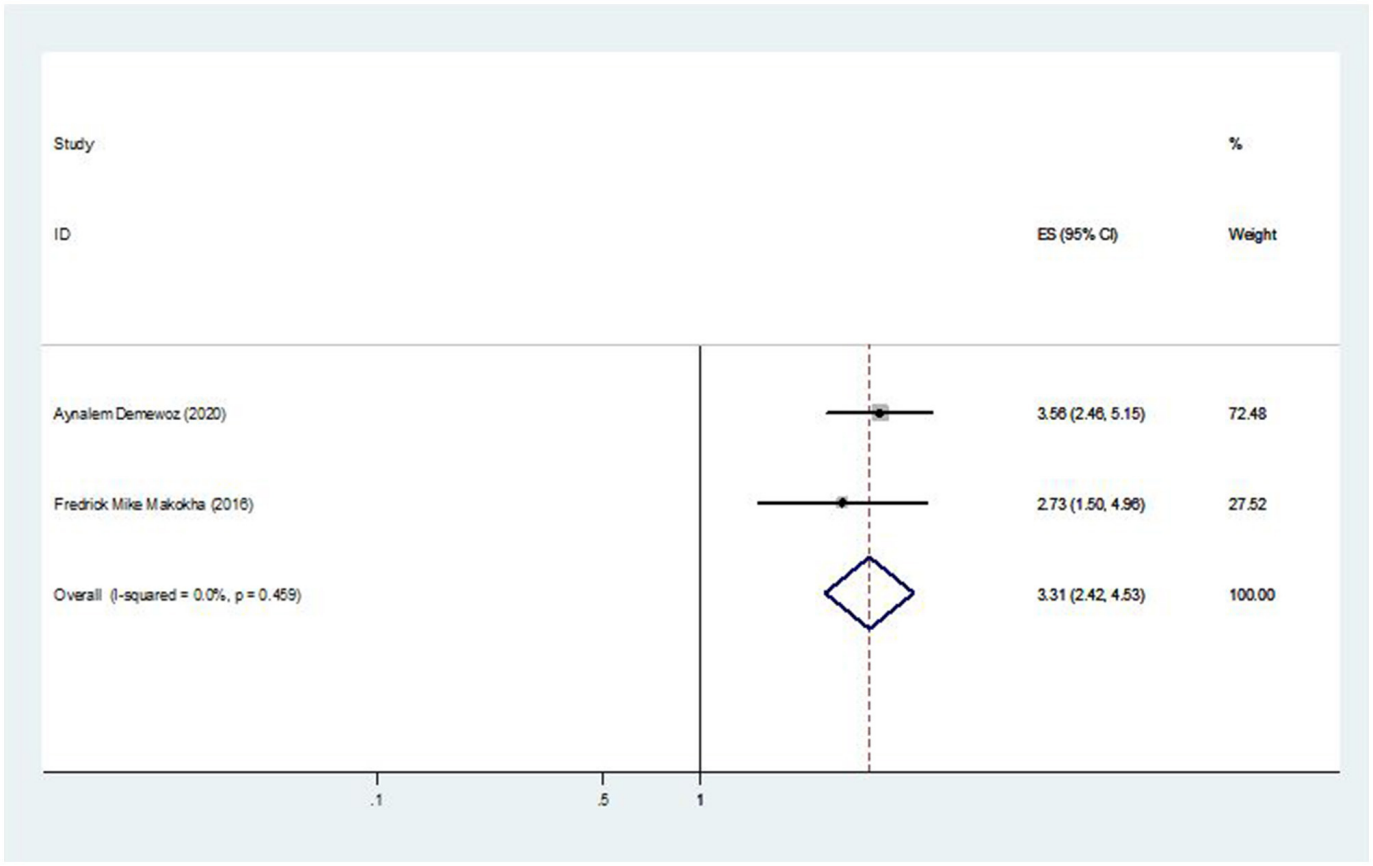
The pooled effect of distance of vaccination site on second-dose measles vaccination coverage in East Africa.

Of the seven studies that were considered, two of them showed an association between the coverage of the second dose of the measles vaccination and the distance from the immunization site. The pooled odds ratio was 3.31 (95% CI: 2.42, 4.53), showing that mothers who live closest to the immunization site are 3.31 times more likely to bring their child for the second dose of the measles vaccination than mothers who have to travel a long distance to receive the vaccination (Figure 10).

## Discussion

To the best of our knowledge, the current meta-analysis is the first of its kind for exploring the second-dose measles vaccination coverage among under-five children in East Africa. Despite employing different strategies and approaches, countries are still having difficulty reaching their vaccine coverage targets, particularly for the second dose of the measles vaccination. This systematic review and meta-analysis study assessed the pooled prevalence of second-dose measles vaccine coverage among under-five children in East Africa. Additionally, birth order, distance of vaccination site, complete immunization for other vaccines, marital status, and information about MCV2 were found to be significantly associated with second-dose measles vaccination coverage in East Africa. Among the limitations of this study is the fact that we only examined cross-sectional research, which can potentially introduce bias into the analysis.

The overall pooled prevalence of second-dose measles vaccination coverage in East Africa was 32.22% [95% CI; (18.82, 45.63)]. The pooled prevalence of this study is consistent within the Africa WHO region 2018 report (25%) (3). However, it is lower than different regions of the WHO in its 2019 report such as Eastern Mediterranean (82.4%), European (91.6%), and Western Pacific (80.7%) (28). Similarly it is lower than the United States (91.5%) (29), South-East Asia Region (80%) (30), and measles vaccination coverage trend in Myanmar from 2014 to 2018; the MCV2 coverage in 2018 was 87% (31). A difference in the vaccine's introduction period and the respondents' sociocultural traits, such as difficulty accessing immunization services, lack of comparably better infrastructure, low socioeconomic position, low literacy rate, and lack of information availability, could be the cause of the low coverage of the second dose of the measles vaccination (32). The other explanation might be that women make different decisions and have poor attitudes and perceptions about vaccinations, which negatively affect the rate of vaccination coverage (33). In order to meet the regional and global targets for the eradication of measles, it will be critical to retain political commitment and assure significant, ongoing investments in addition to increasing the second dose of the measles-containing vaccine.

This study found between-country differences in the second-dose measles vaccination coverage among under-five children in East Africa. The lowest prevalence was observed from Ethiopia (28.15%; 95% CI: 10.63, 45.66) while the highest was in Tanzania (44.20%; 95% CI: 41.12, 47.28). It is very lower than the World Health Assembly (WHA) target to increase routine coverage with the second dose of a measles-containing vaccine, and it is far below (>95%) the second dose of measles coverage than the WHO-recommended coverage for global measles elimination (13). Additionally, there are issues that require extra attention, especially in East Africa where routine vaccinations are taken into account while developing programs. Specific strategies and approaches are required to guarantee access to and appropriate use of immunization services, particularly for the second dose of measles vaccination.

This study found birth order to be a significant determinant of second-dose measles vaccination coverage among under-five children. In this regard, we found that, compared to the first birth order child, children with a higher birth order had a higher likelihood of receiving MCV2. However, it is inconsistent with the finding of a study conducted in China on second-dose measles vaccination (34). Additionally, it contradicts the findings of the study done in East Africa on other types of vaccinations (35), which might be the case because mothers with higher birth orders have firsthand experience of the advantages of immunizations from previous pregnancies and deliveries. Furthermore, compared to their peers, children who had all of the other basic immunizations had a higher chance of receiving MCV2. This finding is due to the possibility that mothers had additional services and health information during their children's earlier vaccinations.

This study also found that mothers who live closest to the immunization site are 3.31 times more likely to bring their child for the second dose of the measles vaccination than mothers who have to travel long distance to receive the vaccination. This finding was consistent with the finding of the study conducted in Shenzhen in East China (36). However, it contradicts the findings of a study conducted in the province of Aceh Jaya, Indonesia (37). The possible reason might be due to mothers who travel very far to bring their children to the vaccination site, their present schedule commitment, and workload from home duties. In addition, it might be due to the fact that majority of people would not travel more than 5 m for basic curative and preventive care. An important factor influencing the usage of healthcare services was distance (38).

Additionally, it was found that, among under-five children, receiving the second dose of the measles vaccination was significantly influenced by them receiving all other recommended vaccinations. In this regard, children who received all other recommended vaccinations were 2.17 times more likely to receive for the second dose of the measles vaccination than children who had not received all other recommended vaccinations. This finding is due to the possibility that mothers had additional services and health information during their children's earlier vaccinations (39). Moreover, mothers may know the routine schedule and the appropriate age for the second-dose vaccination of measles.

The present study also found a significant association between marital status and second-dose measles vaccination coverage. Mothers who are married were 1.47 times more likely to take their child for the second-dose measles vaccination than mothers who are unmarried. Partner involvement has been shown to improve health-seeking behavior and seeking health services (40). One explanation might be that married women receive unfettered emotional and financial support; their spouse might even remind them to get the child vaccinated. Thus, unmarried women can have a disproportionately greater psychological influence, which can affect vaccination uptake.

Moreover, this systematic review and meta-analysis observed that mothers who were aware of the second dose of the measles vaccine were 7.39 times more likely to vaccinate their child than those who were unaware of the second-dose measles vaccination. This finding is consistent with studies from Nepal and India that showed that lack of knowledge of the immunization schedule was the cause of incomplete or partial vaccination (41, 42). This lack of knowledge could be because women who were aware of the vaccination schedule were probably also aware of the benefits of vaccination and the minimum age at which immunizations must be completed. Mothers' intention to vaccinate their children may also be influenced by their increased knowledge of the second dose of the measles vaccine.

## Strengths and limitations

The strengths of this review included a rigorous, standardized methodological approach, broad inclusion criteria, and the involvement of multidisciplinary expertise. Despite prudently extensive search and planned reviews, more than two reviewers minimized all possible risk of bias. The current study is not without limitations. Some of the limitations comprise the fact that we have reviewed only cross-sectional studies that are prone to confounding the number of studies that were not equally distributed among countries. Regarding the intended result, bias may exist, particularly for women without immunization records, and the number of studies included in the current study was very few and may affect the overall result.

## Conclusion

The current study found that the pooled prevalence of second-dose measles vaccine coverage among under-five children was much lower than WHO's target for second-dose measles vaccination coverage and far lower than the prevalence of second-dose measles vaccination coverage across the world. These findings also showed that second-dose measles vaccination among under-five children is affected by birth order, distance of vaccination site, complete immunization for other vaccines, marital status, and information about MCV 2. These factors imply that there is a need for countries and their partners to act urgently to secure political commitment, expand primary health service and health education, and increase vaccination coverage to improve second-dose measles vaccination coverage among under-five children.

## Data availability statement

The raw data supporting the conclusions of this article will be made available by the authors, without undue reservation.

## Author contributions

TA: Conceptualization, Data curation, Formal analysis, Methodology, Software, Validation, Writing – original draft, Writing – review & editing. TT: Formal analysis, Methodology, Software, Writing – review & editing. BW: Conceptualization, Data curation, Formal analysis, Methodology, Writing – review & editing. EM: Conceptualization, Data curation, Formal analysis, Writing – review & editing. MA: Conceptualization, Formal analysis, Methodology, Writing – review & editing. AZ: Data curation, Formal analysis, Software, Writing – review & editing. MW: Formal analysis, Software, Writing – review & editing. AK: Conceptualization, Methodology, Validation, Writing – review & editing. BT: Conceptualization, Methodology, Software, Writing – review & editing. AG: Conceptualization, Formal analysis, Methodology, Validation, Writing – original draft, Writing – review & editing.
